# Physiological Chemistry

**Published:** 1890-11

**Authors:** John A. Miller

**Affiliations:** Professor of Medical Chemistry and Toxicology at the Medical Department of the Niagara University


### PHYSIOLOGICAL CHEMISTRY.

Under the Charge of JOHN A. MILLER, A. M., PH. D.,

Professor of Medical Chemistry and Toxicology at the Medical Department of the Niagara University.

A PTOMAINE EXTRACTED FROM URINE IN A CASE OF "MUMPS." By A. B. Griffiths, (*Chem. News*, 61, 87 ; *Jour. Anal. Chem.*, IV., 351).

In the case referred to, the kidneys were involved, and an alkaloid was extracted by what is called the "ether tartaric acid" or Luff method.

The alkaloid extracted in a case where the parotid and submaxillary glands were both affected, crystallized in white prismatic needles, soluble in water, ether and chloroform. It had a neutral reaction and a slightly bitter taste, and gave precipitates with the general reagents for alkaloids.

The analysis gave results corresponding to the formula :

$$C_6 H_{13} N_3 O_2$$

From its decomposition products, possibly, it may be propylglycocyamine.

This new animal alkaloid is poisonous. Administered to a cat it produced nervous excitement, cessation of the salivary flow, convulsions and death. The alkaloid is not found in normal urines, and the process of its formation is a matter of conjecture.

NOTE ON LEAD POISONING BY CARBONATED BEVERAGES. By D. Woodman, (*Jour. Am. Chem. Soc.*, 11, 99 ; *Jour. Anal. Chem.*, IV., 353).

Attention is called to several cases of poisoning caused by the use of carbonated beverages. The stopper of the bottle was examined and found to consist of a rubber washer, held in place by a metal disk. An analysis of the stopper gave 46 per cent. of lead and 52.2 per cent. of tin.

An analysis of several samples of soda water was made, and they were found to contain from 0.33 to 0.63 grains of lead per gallon. The bottles were kept lying on their sides for one week before opening.

THE PEPTIC AND DIASTATIC FERMENTS OF MICRO-ORGANISMS. By Dr. Claudio Fermi, (*Archiv. für Hygiene*, B. 10, 1890 ; *Jour. Anal. Chem.*, IV., 353).

The following germs produce peptic ferments which dissolve gelatin and fibrin :

1. Anthrax bacillus.
2. Koch's vibrio.
3. Finkler-Prior vibrio.
4. Micrococcus prodigiosus.
5. Micrococcus ascoformis.
6. Bacillus ramosus.
7. B. pyocyanius.
8. Cheese spirillium.
9. Miller's bacillus.
10. B. megaterium.
11. Hay bacillus.
12. Tricophyton tonsurum.

Ferments were isolated from cultures of these germs.

Bacteria which do not liquefy gelatin, produce no peptic ferment.

These ferments were compared with pepsin, trypsin, and papain with the following results :

(1.) Temperature necessary to destroy ferment varies, viz. :

1. M. prodigiosus ............................ 55°C.
2. B. pyocyanius ............................ 60°C.
3. B. anthracis ............................ 65°C.
4. Koch's vibrio ............................ 65°C.
5. Finkler-Prior ............................ 70°C.

(2.) A temperature of 65° destroys the action of pepsin.

(3.) Trypsin heated to 50° no longer digests fibrin, and is without action on gelatin when heated to 60°.

(4.) The ferment of Finkler-Prior and trypsin are alike without effect on fibrin at +4°.

(5.) In presence of five per mille of hydrochloric acid, the ferments of Koch's vibrio, Finkler-Prior, M. prodigiosus, and B. pyocyanius, are without action on fibrin, but still active with gelatin ; under the same conditions trypsin does not act upon either fibrin or gelatin.

(6.) The ferment of anthrax is without action on gelatin in the presence of five per mille of hydrochloric acid.

(7.) Papain and Finkler-Prior ferment alike bear a dry heat of 120°–140°C. without being destroyed.

(8.) The presence of corrosive sublimate one per mille, of salicylic acid, saturated solution, and of carbolic acid five per mille, destroys the activity of the ferments of the cholera germ, the Finkler-Prior bacillus, M. prodigiosus, also of pepsin (salicylic acid excepted) and trypsin on fibrin.

(9.) Of fourteen bacterial ferments only five digest fibrin.

(10.) Egg-albumin, serum-albumin, and diphtheritic membrane are attacked with difficulty by the bacterial ferments.

(11.) Gelatin is acted upon much more readily than fibrin by both trypsin and the bacterial ferments.

(12.) None of the bacterial ferments act upon fibrin in the presence of hydrochloric acid.

The following germs produce very active diastatic ferments:

| | |
|---|---|
| Anthrax. | Miller's bacillus. |
| Cholera. | B. ramosus. |
| Finkler-Prior. | B. Fitz. |
| Cheese spirillium. | B. tetrayenus. |
| B. megaterium. | |
| Hay bacillus. | |

The following produce feeble diastatic ferments:

| | |
|---|---|
| Feces bacillus. | Hay vibrio. |
| B. pyogenes fort. | Steph. cercus et flavus. |
| B. aceticus. | Pneumonia bacillus. |
| B. violanus. | Tricophyton tonsurum. |
| Glanders vacillus. | |

The production of a diastatic ferment by the following is doubtful:

| | |
|---|---|
| Rabbit septicemia. | B. diphtheriticus. |
| B. zopfii. | B. phosphorescens. |
| B. typhosus. | |

Seven ferments were isolated, having the following properties:

(1.) A temperature of 37°C. favors the culture of the diastatic ferments. They are active, however, at +4° and at 50°.

(2.) Heating to 60° destroys the cholera ferment, and to 70° all the others.

(3.) They are not destroyed by a three per cent. solution of carbolic acid, a saturated solution of salicylic acid, and a ten per cent. soda solution, but they are enfeebled by hydrochloric acid five per mille.

(4.) Gum arabic, inulin, amygdalin, and salicin are not affected by bacterial ferments.

(5.) The starches are fermented by the following germs:

| | |
|---|---|
| 1. B. Fitz. | 6. Cheese spirillium. |
| 2. B. megaterium. | 7. B. violaceus. |
| 3. B. Miller. | 8. B. pyog. fort. |
| 4. Cholera germ. | 9. B. tetrayenus. |
| 5. Finkler-Prior. | |

(6.) Hay bacillus and b. ramosus change starch into sugar without causing further fermentation.

(7.) Both peptic and diastatic ferments may be produced by one and the same germ.

Poisonous Products of Sapogenous Intestinal Bacteria. By Bagiknsky and Startthagen, (*Berlin Klinische Wochens.*, No. 13, 1880 ; *Jour. Analy. Chem.*, IV., 358).

The white, liquefying bacillus, which is frequently found in the diarrheal stools of infants, was grown at 35°C. for ten days on sterilized broth made from horse flesh. A poisonous base was obtained, which agrees very closely with the one obtained by Brieger from meat, which had been undergoing ordinary putrefaction for four months. There was also obtained a poisonous peptone, which caused death in from forty-eight to seventy-two hours. Heated to 100°C. enfeebled this peptone, but did not wholly destroy its poisonous properties.

When grown in sterilized milk, this same germ does not produce the basic substance mentioned, but after keeping for three days at 35°C. the unfiltered milk is intensely poisonous. If the milk cultures be kept until complete coagulation takes place, the poisonous properties will be found to have disappeared. The peptone prepared from the milk seems to be quite different from that obtained from the meat cultures.

Proteids of Liver and Kidney Cells. By W. D. Halliburton, (*Proc. Physiol. Soc.*, 1890, 7 ; *Jour. Chem. Soc.*, Sept., 1890).

The proteids obtained of these cells belong almost exclusively to the class of globulins. The nucleo-albumin, which was previously described in lymph cells, is absent from liver cells, but present in kidney cells. No evidence of true myosin or myosinogen is found in either, although portions of the flesh tissues are found to hasten the coagulation of salted blood plasma, and of hydrocele and pericardial fluids very considerably. This power is destroyed at a fairly low temperature, and attempts to prepare fibrin ferment from the cells by Schmidt's alcohol method failed.

Is Free Hemoglobin Present in the Blood-Plasma of the Splenic Vein? By E. A. Schaefer, (*Proc. Physiol. Soc.*, 1890, 9-10 ; *Jour. Chem. Soc.*, September, 1890).

The observations were made for the purpose of testing the statements which occur in many text-books of physiology, (the origin of which appears to be obscure,) that the blood-plasma of the splenic vein normally contains free hemoglobin. The investigation, covering a period of two years, of the blood of a large number of animals of different kinds, killed at various stages of digestion proved that free hemoglobin is normally absent both in the blood-plasma of the splenic vein and in that of arterial blood.

Lecithin and Cholesterin in Red Blood Corpuscles. By P. Manasse, (*Zeit. Physiol. Chem.*, 14-437 ; *Jour. Chem. Soc.*, September, 1890).

The cholesterin in the red corpuscles of the blood is identical with that obtained from gallstones. The corpuscles contain as a mean 0.151 per cent. of cholesterin. The lecithin is identical with that obtained from egg-yolk, brain, etc. The corpuscles contain as a mean 1.867 per cent. of lecithin.

Pernicious Anemia. By W. Hunter, (*Brit. Med. Jour.*, 1890, II., 1-4, 81-85 ; *Jour. Chem. Soc.*, September, 1890).

A case of this disease is described with clinical details. The urine was normal in quantity, of low specific gravity, and contained as its principal pigment "pathological urobilin." Occasionally cells of renal origin, containing blood-pigment, were present. The urine contained *excess of iron.* The average secretion of iron in the urine in health is from three to five milligrams daily ; in three cases of chlorosis it averaged 1.76 milligrams ; in the present case it varied from 6.5 to 32.3 milligrams. The liver was found to be richer in iron than normal.

The opinion was formerly expressed that the destruction of the blood corpuscles, characteristic of the disease, which occurs in the portal circulation, and which is similar in essential characteristic to that produced by poisoning with toluylenediamine, is probably brought about by certain poisonous agents of a cadaveric nature absorbed from the intestinal tract. Special attention was, therefore, directed to the products of putrefaction in the urine. Estimation of the etherial hydrogen sulphates and their ratio to the normal sulphates showed that the absolute amount of putrefaction, as measured by that of the aromatic sulphates, was not increased ; the normal ratio of the two classes of sulphates was, however, considerably altered, being 1:3 instead of the normal 1:10. Improvement in the patient's condition were coincident with the returns to the normal ratio.

It was further found that the urine contained a *ptomaine*, putrescine (tetramethylenediamine), a substance absent in the urine of health and that of most diseased conditions, but present in that of cystinuria and probably also in that of cases of cholera. In one specimen another diamine, which was not fully identified, was also discovered. These diamines are not, however, markedly toxic, and they can hardly be considered the poisons that produce the blood-destruction, but their presence indicates the existence of certain special microörganisms in the alimentary tract, and, therefore, a condition more than any other favorable to the production of other and more poisonous alkaloids not yet discovered.

A further support to the supposition that the alimentary tract is the seat of the formation of the poison, was found in the fact that the gastro-intestinal mucous membrane was distinctly unhealthy in appearance, showing well-defined patches of inflammation, resembling those frequently seen in the kidney when that organ is the seat of localized infection with pathogenic microbes. The neighboring lymphatic glands were similarly affected.

